# Efficacy, safety, and immunogenicity of proposed biosimilar RGB-19 and tocilizumab intravenously administered to adults with active rheumatoid arthritis and an inadequate response to methotrexate: a phase 3, randomised study

**DOI:** 10.1016/j.ero.2025.12.010

**Published:** 2026-01-27

**Authors:** Hiroaki Matsuno, Ernest Choy, Paul Emery, Masato Okada, Roshan Dias, Károly Horvát-Karajz, Gordana Dancer, Yusuke Karibe, Suguru Masuda, Joachim Kiefer, Gerd R Burmester

**Affiliations:** 1 Matsuno Clinic for Rheumatic Disease, Toyama, Japan; 2 Immuno-Rheumatology Center, St. Luke's International Hospital, Tokyo, Japan; 3 Division of Infection and Immunity, CREATE Centre, Cardiff University, Cardiff, UK; 4 Leeds NIHR Biomedical Research Centre, Leeds Teaching Hospitals Trust and Leeds Institute of Rheumatic and Musculoskeletal Medicine, University of Leeds, Leeds, UK; 5 Immuno-Rheumatology Centre, St. Luke's International Hospital, Tokyo, Japan; 6 Gedeon Richter Plc, Budapest, Hungary; 7 Mochida Pharmaceutical Co., Ltd, Tokyo, Japan; 8 Department of Rheumatology and Clinical Immunology, Charité—Universitätsmedizin Berlin, Berlin, Germany

## Abstract

**Objectives:**

This phase 3 study (jRCT2031220512) evaluated the equivalence in efficacy, and compared the serum concentration, pharmacodynamics, safety, and immunogenicity of proposed biosimilar RGB-19 and tocilizumab in adults with active rheumatoid arthritis (RA).

**Methods:**

Participants were randomly assigned 1:1 to intravenous infusions of 8 mg/kg RGB-19 or tocilizumab every 4 weeks, with efficacy evaluated to week 52 and safety follow-up to week 54. The primary endpoint was the mean change from baseline (CfB) in Disease Activity Score based on 28 joints with erythrocyte sedimentation rate (DAS28-ESR), with equivalence determined if the 2-sided 95% CI for the pooled difference between groups was within the predefined equivalence margin (±0.6 at week 12). Secondary objectives were other efficacy indicators, serum drug concentration, pharmacodynamics, safety, and immunogenicity.

**Results:**

In total, 368 participants were randomly assigned to treatment (RGB-19 N = 182; tocilizumab N = 186). At week 12, the difference in mean DAS28-ESR CfB between groups (RGB-19 n = 173; tocilizumab n = 181) was -0.21 (95% CI: -0.43, 0.02; within the equivalence margin). All other efficacy indicators and serum drug concentration values were similar between groups up to week 52. Median absolute neutrophil count, C-reactive protein, and soluble interleukin-6 receptor values were similar between groups to week 52. Safety profiles were similar, with a similar incidence of treatment-emergent adverse events and adverse drug reactions to week 54. The incidence of treatment-emergent antidrug and neutralising antibodies was similarly low in both groups.

**Conclusions:**

This study demonstrated equivalent efficacy, and similar safety and immunogenicity, of RGB-19 and reference tocilizumab for participants with active RA.

**WHAT IS ALREADY KNOWN ON THIS TOPIC**

• Tocilizumab is a biologic with proven efficacy in numerous inflammatory diseases; however, high treatment costs can limit patient access. Tocilizumab biosimilars can provide an equivalent therapy at a reduced cost, with the potential to expand treatment access.

**WHAT THIS STUDY ADDS**

• In this phase 3 study, the proposed biosimilar RGB-19 demonstrated equivalent efficacy, and similar safety and immunogenicity to reference tocilizumab in participants with active rheumatoid arthritis and an inadequate response to methotrexate.

**HOW THIS STUDY MIGHT AFFECT RESEARCH, PRACTICE OR POLICY**

• These data demonstrate the biosimilarity in terms of efficacy of RGB-19 and tocilizumab. RGB-19 has the potential to provide an alternative to tocilizumab with the same therapeutic benefit at a lower cost.

Alt-text: Unlabelled box dummy alt text

## INTRODUCTION

Rheumatoid arthritis (RA) is an inflammatory disease with interleukin-6 (IL-6) involvement [1], a cytokine that is crucial for both innate and adaptive immunity [2]. IL-6 plays a key role in the inflammatory processes in RA, with elevated levels of both IL-6 and IL-6 receptor (IL-6R) found in the serum and synovial fluid of affected joints [1].

Tocilizumab is a humanised anti-human IL-6R antibody that competitively inhibits IL-6 signalling by blocking the IL-6 binding site of IL-6R [3,4]; it was approved for the treatment of RA in patients with an inadequate response to previous therapy in Japan in 2008 [5], Europe in 2009 [6], and the United States in 2010 [7]. As well as for RA, tocilizumab is licenced for a wide range of inflammatory disorders, including juvenile idiopathic arthritis, systemic juvenile idiopathic arthritis, and giant cell arteritis [2,8,9]. The efficacy [[10], [11], [12], [13], [14], [15], [16], [17], [18]] and tolerability [14,19,20] of tocilizumab for the treatment of RA have been demonstrated in numerous studies, and it was the first humanised anti-IL-6R monoclonal antibody approved for the treatment of RA [21]. It can be administered as both intravenous (IV) and subcutaneous (SC) formulations [6].

The relatively high cost of biological disease-modifying antirheumatic drugs (DMARDs), such as tocilizumab, compared with alternative treatments may limit patient access [22,23]. Biosimilars have the same mode of action and a similar efficacy and safety profile to originator products with reduced cost, which may help to improve patient access [24]. Biosimilars have been developed for use in RA [[25], [26], [27], [28]]; for example, comparable safety and efficacy of etanercept, adalimumab, and infliximab biosimilars have been demonstrated compared with their reference products [27,29,30].

RGB-19 is a proposed biosimilar [6,31] with the same dosage forms, strength, and route of administration as reference tocilizumab [6]. A phase 1 study comparing a single SC administration of RGB-19 and tocilizumab demonstrated bioequivalence in pharmacokinetics (PK) and similarity in pharmacodynamics (PD), immunogenicity, and safety outcomes [32].

Building on phase 1 evidence for the equivalence in PK/PD and safety with an SC formulation, the aim of this phase 3 (jRCT2031220512) study was to assess outcomes in patients with RA treated with an IV formulation of RGB-19. The study evaluated the equivalence in efficacy, and compared the serum concentration, PD, safety, and immunogenicity of IV infusions of RGB-19 and European Union-licenced tocilizumab (hereafter tocilizumab) in Japanese adults with active RA and an inadequate response to methotrexate.

## METHODS

### Study design

This was a phase 3, randomised, double-blind, multicentre study conducted in Japan (Supplementary Fig S1). Participants were randomly assigned 1:1 to IV infusions of RGB-19 or tocilizumab at a dose of 8 mg/kg every 4 weeks, with oral methotrexate at a dose of 6 to 16 mg/wk as an essential concomitant medication. Randomisation was performed using an investigational product allocation table created via the permuted block method with the medical institution as a block, according to the allocation specifications. Treatments were allocated by the responsible person using the investigational product allocation table, and participants were randomly assigned to one of the treatment groups in a 1:1 ratio via an electronic-based interactive web response system.

Efficacy was evaluated to week 52, and safety was assessed to week 54. The screening period ran from day -42 (week -6) to day -1, the primary evaluation period ran from week 0 to week 12, and the secondary evaluation period ran from week 12 to week 52 or discontinuation. Visits occurred every 4 weeks except for an additional visit at week 2. Participants did not receive the investigational product (IP) at week 2 (visit 3) or week 52 (visit 16). Participants with unresolved serious adverse events (SAEs) or unresolved related adverse events (AEs) by the end of the study were followed up until recovery. For participants who discontinued during the primary evaluation period, safety follow-up for AEs and concomitant therapies was conducted 6 weeks after the last administration of IP. However, if participants who discontinued or withdrew from the study during the primary evaluation period had not recovered by the end of this period or the end of safety follow-up, whichever was longer, follow-up investigation was conducted. For participants who withdrew from the study during the secondary evaluation period, the scheduled date of the final visit was 14 days after completion of all observations, tests, and assessments at the time of discontinuation or 42 days after the last administration of the IP, whichever was later.

Due to time constraints as a result of the COVID-19 pandemic, no patients were involved in setting study research questions or outcome measures, or in developing plans for recruitment, design, or implementation of the study. No patients were asked to advise on the interpretation or writing of results.

### Study population

The study enrolled participants aged 20 to 75 years with active RA. Participants’ RA was classified as functional class I, II or III, according to American College of Rheumatology (ACR) criteria, with active RA defined as a Disease Activity Score based on 28 joints with erythrocyte sedimentation rate (DAS28-ESR) of ≥3.2, swollen joint counts ≥6 (out of total 66), tender joint counts ≥6 (out of 68), an ESR rate ≥28 mm/h or C-reactive protein (CRP) ≥1.0 mg/dL. Participants were also required to have an inadequate response to methotrexate administered for at least 12 weeks before the granting of informed consent and to be on a stable dose of methotrexate 6 to 16 mg/wk for at least 4 weeks before baseline.

Participants were excluded if they met any of the following criteria: body weight of <30 kg or >100 kg; previous treatment with ≥2 biological DMARDs or biosimilar products; previous treatment with tocilizumab or other IL-6 inhibitors or IL-6R inhibitors, conventional synthetic DMARDs (excluding oral methotrexate and leflunomide) within 4 weeks of baseline, or Janus kinase inhibitors. Full exclusion criteria are included in the Supplementary Methods. Changes to methotrexate administration were prohibited except for safety reasons.

### Study endpoints

The primary endpoint was the mean change from baseline (CfB) in DAS28-ESR score at week 12. RGB-19 demonstrated equivalent efficacy to tocilizumab if the 2-sided 95% CI for the pooled difference between treatment groups was included in the predefined equivalence margin of ±0.6.

The key secondary efficacy endpoints were mean CfB in DAS28-ESR score and remission rates for DAS28-ESR, Clinical Disease Activity Index (CDAI), Simplified Disease Activity Index (SDAI) and European Alliance of Associations for Rheumatology (EULAR) at baseline (week 0), and weeks 8, 12, 16, 24 and 52, and the proportion of participants achieving ACR20, ACR50, and ACR70 at weeks 8, 12, 16, 24, and 52. Mean CfB in DAS28-CRP was calculated as a post-hoc analysis, following database lock.

Serum drug concentration was assessed at baseline (week 0), and weeks 2, 4, 12, 24, 36, and 52. To assess PD, absolute neutrophil count (ANC), CRP, and soluble IL-6 receptor (sIL-6R) levels were assessed at baseline (week 0) and weeks 12, 24, and 52. Safety analyses included the assessment of AEs and immunogenicity findings (antidrug antibody [ADA] positivity, titre, and neutralising antibodies [NAbs]) over the study period.

### Data analyses

Based on a predicted dropout rate from the full analysis set (FAS) of 2%, a target sample size of 358 randomly assigned participants (179 per group) was used.

FAS was defined as the primary analysis set and included randomly assigned participants who were administered at least 1 infusion of the IP and had a DAS28-ESR score at baseline and at least 1 postbaseline assessment. The statistical analysis was performed using SAS v9.4. In the primary analysis, the difference in CfB in DAS28-ESR at week 12 between the RGB-19 and tocilizumab groups was calculated using analysis of covariance (ANCOVA), with treatment group and the presence/absence of administration history of biological DMARDs included as factors and DAS28-ESR score at baseline as a covariate. If DAS28-ESR was missing due to the occurrence of an intercurrent event, DAS28-ESR at each time point (baseline, weeks 8 and 12) was imputed using the multiple imputation method under the assumption of missing at random. The procedure for imputation of missing data and pooling the analysis results after imputation was that only nonmonotonic missing data were imputed based on multiple imputation using Markov Chain Monte Carlo by treatment group. The number of imputations was 100. Data obtained from this imputation, including monotone missing data, were imputed based on multiple imputation using the monotone regression method by treatment group. The number of imputations was 1. For the 100 pseudocomplete data generated, the ANCOVA model was used to estimate the difference between groups independently for each dataset. One hundred analysis results were pooled according to Rubin’s rule [33]. To confirm the robustness of the results from the primary analysis, a tipping point sensitivity analysis was performed (Supplementary Methods).

The PK analysis set included randomly assigned participants who were administered at least 1 infusion of the IP, had at least 1 serum drug concentration result after IP administration and had no major protocol deviations that could have an impact on the results of the PK measure. The PD analysis set included randomly assigned participants who were administered at least 1 infusion of the IP, had at least 1 PD result after IP administration, and had no major protocol deviations that could have an impact on the results of the PD measure.

The safety analysis set included participants who, during the screening period, were administered at least 1 essential concomitant medication and had safety assessment data and participants who, during the primary and secondary evaluation period, were administered at least one infusion (partial or full dose) of IP and had postbaseline safety assessment data. Safety data were analysed descriptively and summarised by the number of events and the number/percentage of participants experiencing events. A treatment-emergent AE (TEAE) was defined as an AE that occurred on or after the first IP administration. Significant AEs were defined as AEs that led to IP discontinuation and AEs of special interest (AESIs).

The immunogenicity analysis set included participants who were administered at least 1 infusion (partial or full dose) of IP, had no protocol deviations that could have an impact on immunogenicity results, had a predose immunogenicity result, and at least 1 available postdose immunogenicity assessment. Presence of ADAs and NAbs and the titre, number, and percentage of participants with ADAs and NAbs were summarised by treatment group and timepoint.

A participant was considered to have completed the study if they had completed all study periods, including the last visit at week 54. Descriptive statistics were provided by treatment group for continuous endpoints. For binary variables, percentages were calculated for each treatment group.

### Ethics

The institutional review boards that reviewed and approved the study protocol before the start of the study are listed in the Supplementary Material. The conduct of this clinical study met all local legal and regulatory requirements and was conducted in line with the Declaration of Helsinki. Each participant provided written informed consent.

## RESULTS

### Participant population

**Figure 1:**
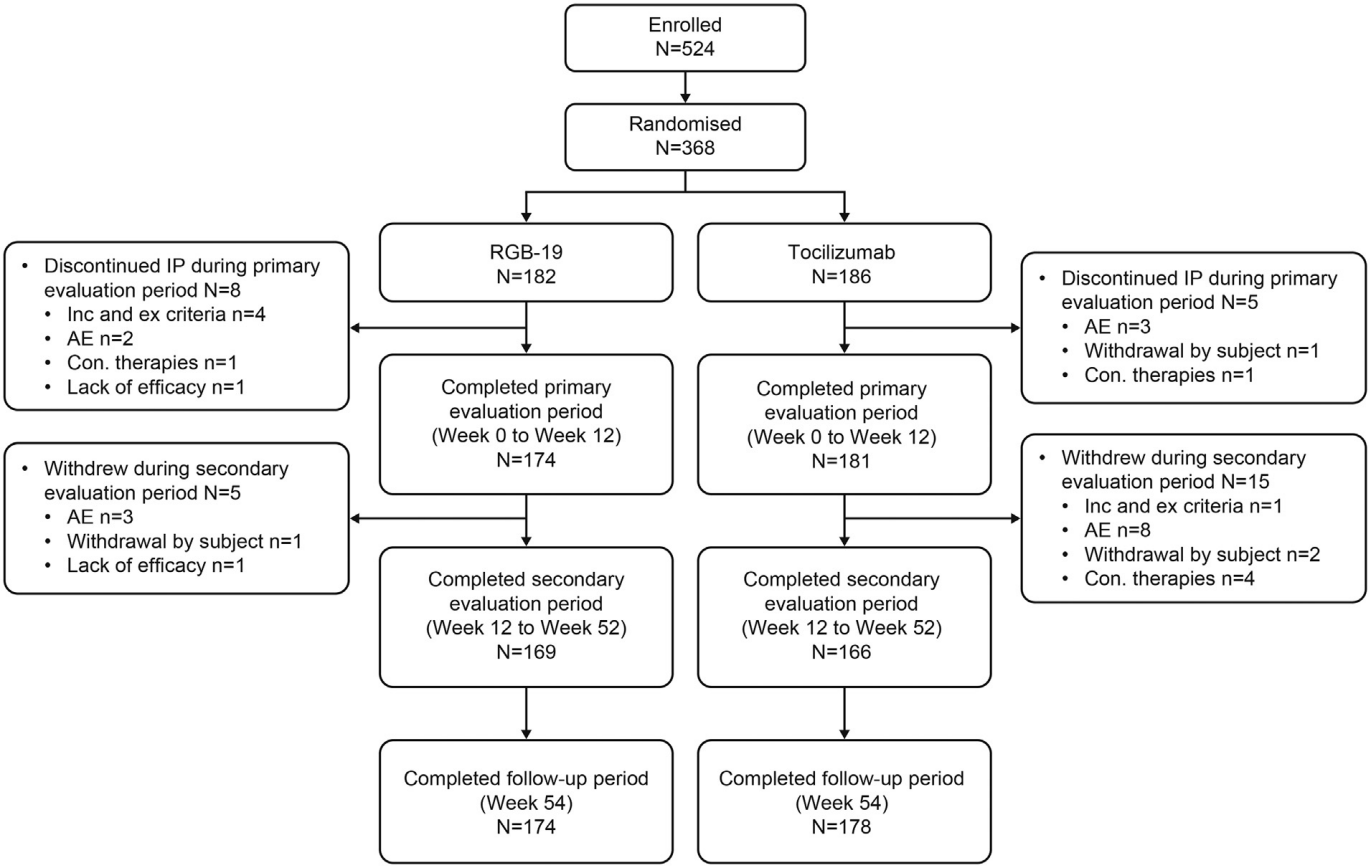
Participant disposition flow chart. AEs, adverse events.

In total, 368 participants were randomly assigned to receive study treatment (RGB-19 N = 182; tocilizumab N = 186). Overall, 355 completed the primary evaluation period (RGB-19 n = 174; tocilizumab n = 181), and 335 completed the secondary evaluation period (RGB-19 n = 169; tocilizumab n = 166). The most common reason for withdrawal or discontinuation in the primary and secondary evaluation periods was due to AEs (RGB-19 n = 5; tocilizumab n = 11) (Fig 1).

### Baseline demographics and characteristics

Baseline characteristics were similar between the 2 treatment groups (Table 1). All participants were of Asian race, median age (range) was 57 (20-75) years, and most were female (75.8%) with class II RA (63.3%; Table 1). The overall median (25th percentile-75th percentile) duration since first RA diagnosis was 2.95 (0.65-8.91) years and was similar for both treatment groups (RGB-19: 3.22 [0.82-8.74] years; tocilizumab: 2.63 [0.59-9.14] years; Table 1). There were 171 (94.0%) participants in the RGB-19 group and 169 (90.9%) participants in the tocilizumab group who had concomitant diseases. In both the RGB-19 and tocilizumab groups, the most frequently occurring concomitant diseases were within the system organ class metabolism and nutrition disorders (70 [38.5%] and 62 [33.3%] participants, respectively; Supplementary Table S1). The mean (SD) dose of methotrexate (an essential concomitant medication in this study) at baseline was similar between groups (RGB-19: 9.6 [2.7] mg/wk; tocilizumab: 9.6 [2.6] mg/wk; Table 1). Overall, 69 participants (18.8%) had a history of having been administered biological DMARDs, with the majority being tumour necrosis factor-α-inhibitors (55 [14.9%]), of which etanercept was the most common (22 [6.0%]; Supplementary Table S1). During the study, the most frequently used restricted concomitant medications (defined as nonsteroidal anti-inflammatory, analgesic drugs [except for strong opioids, which were prohibited], corticosteroids, hyaluronan by intra-articular administration, and arthrocentesis), by Anatomical Therapeutic Chemical 4 class, were topical nonsteroidal anti-inflammatory drug preparations, glucocorticoids, and propionic acid derivatives. The number of patients receiving glucocorticoids as concomitant medication was balanced between groups (RGB-19: 44%; tocilizumab: 42.5%).

**Table 1:** Baseline demographics (FAS)

Characteristics	RGB-19 (N = 182)	Tocilizumab (N = 186)	Total (N = 368)
Female, n (%)	141 (77.5)	138 (74.2)	279 (75.8)
Age (y)			
Mean (SD)	56.7 (12.4)	55.4 (12.7)	56.0 (12.6)
Body weight (kg) (baseline)			
Mean (SD)	56.9 (12.3)	58.5 (13.6)	57.7 (13.0)
RF test result (IU/mL) (screening period)			
Mean (SD)	151.6 (403.2)	189.3 (568.7)	170.7 (493.5)
Positive, n (%)	159 (87.4)	159 (85.5)	318 (86.4)
Anti-CCP antibody level (U/mL) (screening period)			
Mean (SD)	343.4 (400.2)	355.8 (431.5)	349.6 (415.8)
Positive, n (%)	162 (89.0)	161 (86.6)	323 (87.8)
Duration from first diagnosis of RA (y)^a^			
Median (25th percentile-75th percentile) [range]	3.22 (0.82-8.74) [0.2-46.3]	2.63 (0.59-9.14) [0.2-37.2]	2.95 (0.65-8.91) [0.2-46.3]
Functional classification of RA, n (%)			
Class I	43 (23.6)	42 (22.6)	85 (23.1)
Class II	117 (64.3)	116 (62.4)	233 (63.3)
Class III	22 (12.1)	28 (15.1)	50 (13.6)
Class IIII	0 (0.0)	0 (0.0)	0 (0.0)
Dose of MTX (mg/wk) (baseline)			
Mean (SD)	9.6 (2.7)	9.6 (2.6)	9.6 (2.7)
DAS28-ESR, mean (SD)	6.2 (0.9)	6.1 (0.9)	6.1 (0.9)
CDAI, mean (SD)	34.6 (11.6)	33.6 (11.4)	34.1 (11.5)
SDAI, mean (SD)	36.7 (12.5)	35.6 (12.2)	36.1 (12.3)
TJC68, mean (SD)	16.1 (9.7)	16.4 (10.0)	16.3 (9.8)
SJC66, mean (SD)	14.2 (7.5)	13.8 (7.3)	14.0 (7.4)
ESR (mm/h), mean (SD)	45.9 (21.3)	45.2 (24.4)	45.6 (22.9)
CRP (mg/dL), mean (SD)	2.0 (2.3)	2.0 (2.6)	2.0 (2.4)
Evaluator's Global Assessment (EGA) (mm), mean (SD)	64.7 (20.3)	61.7 (19.3)	63.2 (19.8)
PGA (mm), mean (SD)	63.9 (21.0)	60.0 (22.4)	61.9 (21.7)
PtAP (mm), mean (SD)	65.2 (21.2)	61.9 (22.6)	63.6 (21.9)
HAQ-DI, mean (SD)	1.1 (0.7)	1.1 (0.6)	1.1 (0.7)

CCP, cyclic citrullinated peptide; CDAI, Clinical Disease Activity Index; CRP, C-reactive protein; DAS28-ESR, Disease Activity Score in 28 joints-Erythrocyte Sedimentation Rate; DMARDs, disease-modifying antirheumatic drugs; EGA, evaluator's global assessment; ESR, erythrocyte sedimentation rate; FAS, full analysis set; HAQ-DI, Health Assessment Questionnaire–Disability Index; MTX, methotrexate; NSAID; nonsteroidal anti-inflammatory drug; PGA, patient global assessment; PtAP, patient's assessment of pain; RA, rheumatoid arthritis; RF, rheumatoid factor; SDAI, Simplified Disease Activity Index; SJC, swollen joint count; TJC, tender joint count.

a All enrolled participants had a disease duration of ≥12 wk, and no cases violated inclusion criteria.

### CfB in DAS28-ESR

**Figure 2:**
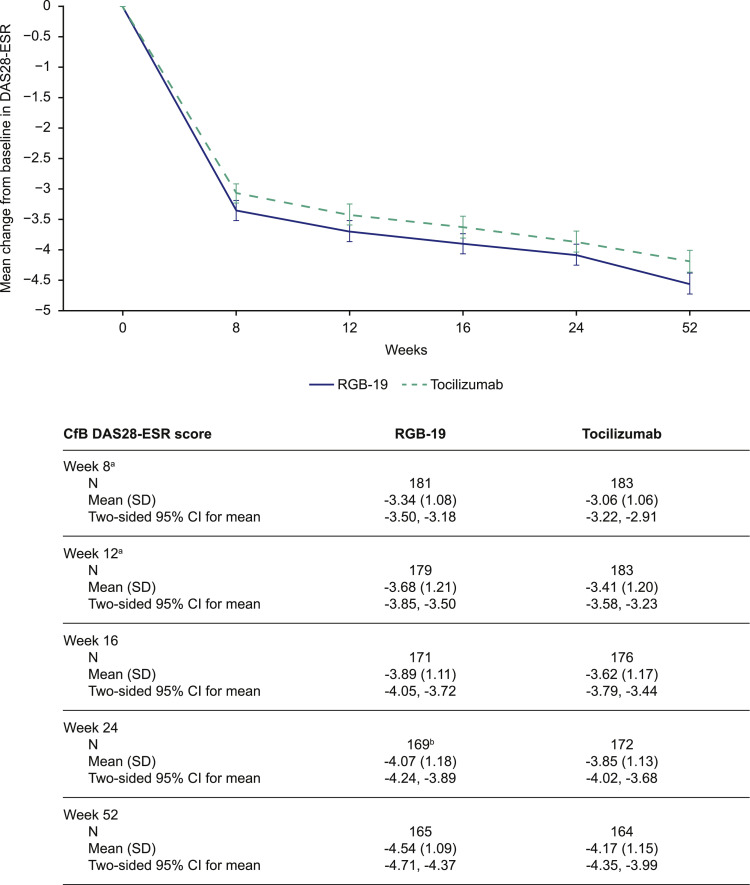
DAS28-ESR score and mean change from baseline from week 8 to week 52 (FAS). Error bars depict the 95% CI; ^a^includes measurements after occurrence of intercurrent events; ^b^for 1 participant ESR (mm/h) was not measured at week 24, but the other components were measured. Therefore, DAS28-ESR could not be calculated and was missing for this participant. CfB, change from baseline; CI, confidence interval; DAS28-ESR, Disease Activity Score in 28 joints-Erythrocyte Sedimentation Rate; FAS, full analysis set.

At week 12, the primary endpoint of the adjusted mean (SE) values was similar for both groups (RGB-19: -3.62 [0.09], n = 173 [observed cases]; tocilizumab: -3.41 [0.09], n = 181 [observed cases]). The adjusted mean difference between the groups was -0.21 (95% CI: -0.43 to 0.02), which fell within the margin of ±0.6 and so demonstrated the equivalence in efficacy between RGB-19 and tocilizumab. The results of the sensitivity analyses were supportive of the primary analysis (Supplementary Table S2, Supplementary Fig S2). The mean CfB in DAS28-ESR score was similar between treatment groups at each timepoint up to week 52 (Fig 2). The CfB in DAS28-CRP was also similar between groups at week 12 (adjusted mean [SE] RGB-19: -2.93 [0.08], tocilizumab: -2.76 [0.08]; adjusted mean difference point estimate: -0.18 [95% CI: -0.37 to 0.02]).

### Remission and achievement rate endpoints

Remission rates (CDAI, DAS28, and SDAI) were similar between groups up to week 52 with remission rates increasing for both groups over time (Table 2). EULAR response rates were approximately 100% at all timepoints, following the first administration in both groups (Table 2). The proportion of participants achieving ACR20, 50, or 70 was similar between RGB-19 and tocilizumab up to week 52, with rates increasing over time (Table 2).

**Table 2:** CDAI, DAS28, SDAI, and EULAR efficacy endpoints and remission/response rates and achievement of ACR20/50/70 (FAS)

Efficacy remission/response rates and achievement endpoints	CDAI ≤2.8^a^			SDAI ≤3.3^a^		DAS28 <2.6^a^		EULAR^a^		ACR20^a^		ACR50^a^		ACR70^a^	
	RGB-19	Tocilizumab		RGB-19	Tocilizumab	RGB-19	Tocilizumab	RGB-19	Tocilizumab	RGB-19	Tocilizumab	RGB-19	Tocilizumab	RGB-19	Tocilizumab
Week 8															
N	176	181		176	181	176	181	176	181	176	181	176	181	176	181
Remission rate (%)	9.7	5.5		10.8	5.5	44.9	38.7	100.0	99.4	85.2	77.3	51.1	45.9	26.7	18.2
Difference PE (95% CI)	4.1 (-1.5 to 10.0)			5.3 (-0.5 to 11.3)		6.2 (-4.0 to 16.2)		0.6 (-1.6 to 3.1)		7.9 (-0.2 to 15.9)		5.3 (-5.0 to 15.4)		8.5 (-0.2 to 17.0)	
Week 12															
N	177	182		177	182	177	182	177	182	177	182	177	182	177	182
Remission rate (%)	18.6	13.7		22.0	15.9	56.5	53.3	99.4	100.0	87.6	85.7	63.8	61.5	34.5	31.3
Difference PE (95% CI)	4.9 (-2.7 to 12.6)			6.1 (-2.0 to 14.2)		3.2 (-7.0 to 13.3)		-0.6 (-3.1 to 1.6)		1.9 (-5.3 to 9.0)		2.3 (-7.6 to 12.2)		3.1 (-6.5 to 12.8)	
Week 16															
N	171	176		171	176	171	176	171	176	172	176	172	176	172	176
Remission rate (%)	20.5	23.3		22.8	26.7	63.7	58.0	100.0	99.4	91.9	85.2	71.5	66.5	43.0	45.5
Difference PE (95% CI)	-2.8 (-11.5 to 5.9)			-3.9 (-12.9 to 5.2)		5.8 (-4.5 to 15.8)		0.6 (-1.7 to 3.1)		6.6 (-0.1 to 13.4)		5.0 (-4.7 to 14.6)		-2.4 (-12.7 to 7.9)	
Week 24															
N	177	182		177	182	177	182	177	182	177	182	177	182	177	182
Remission rate (%)	28.2	24.2		32.2	28.0	66.7	65.4	99.4	98.9	94.4	90.7	71.8	72.5	51.4	50.5
Difference PE (95% CI)	4.1 (-5.0 to 13.1)			4.2 (-5.3 to 13.6)		1.3 (-8.5 to 11.0)		0.5 (-2.2 to 3.4)		3.7 (-1.9 to 9.4)		-0.8 (-10.0 to 8.4)		0.9 (-9.4 to 11.1)	
Week 52															
N	177	182		177	182	177	182	177	182	177	182	177	182	177	182
Remission rate (%)	44.6	38.5		49.7	40.7	79.7	73.1	99.4	98.9	96.0	92.3	84.2	79.7	67.8	61.0
Difference PE (95% CI)	6.2 (-4.0 to 16.2)			9.1 (-1.2 to 19.1)		6.6 (-2.2 to 15.2)		0.5 (-2.2 to 3.4)		3.7 (-1.3 to 9.0)		4.5 (-3.5 to 12.4)		6.8 (-3.1 to 16.5)	

ACR, American College of Rheumatology; CDAI, Clinical Disease Activity Index; DAS28, Disease Activity Score in 28 joints; EULAR, European Alliance of Associations for Rheumatology; FAS, full analysis set; LOCF, last observation carried forward; NRI, nonresponder imputation; OC, observed case; PE, point estimate; SDAI, Simple Disease Activity Index.

a Imputation method OC (LOCF for weeks 12, 24, and 52).

### Secondary drug concentration and PD endpoints

**Figure 3:**
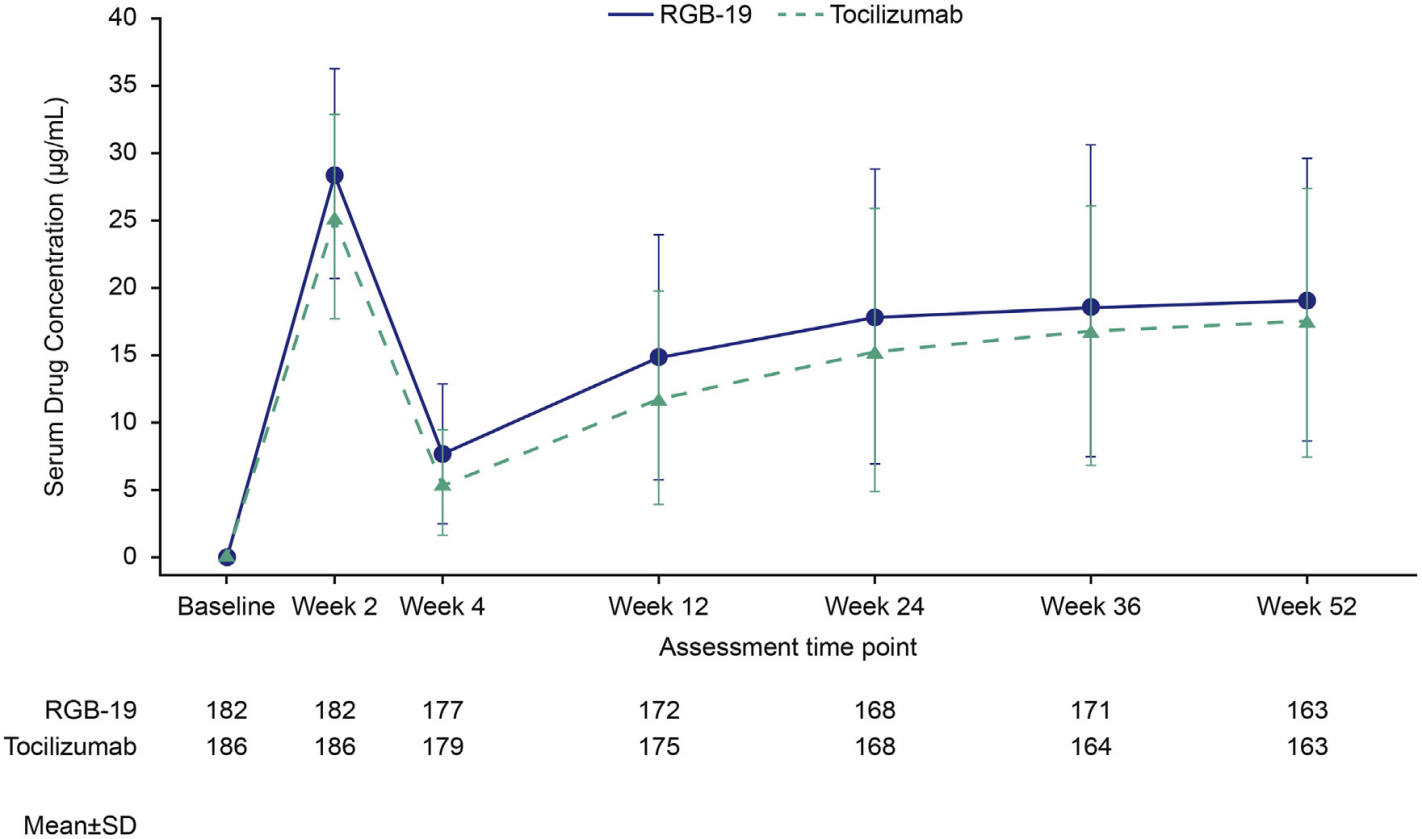
Serum drug concentration from baseline to week 52 in participants receiving RGB-19 or tocilizumab (PK analysis set). PK, pharmacokinetics.

Serum drug concentration values were similar between groups at all timepoints assessed up to week 52 (Fig 3). ADA status was found to have no impact on serum drug concentration; ADA-negative participants had similar serum drug concentration at week 52 (RGB-19: 19.2 [SD 10.6] μg/mL, tocilizumab: 17.4 [SD 10.0] μg/mL) compared with ADA-positive participants (RGB-19: 16.0 [SD 7.6] μg/mL, tocilizumab: 18.6 [SD 10.2] μg/mL). Median ANC, CRP, and sIL-6 receptor values were similar for both treatments for all timepoints up to week 52. Median ANC and CRP levels decreased from baseline and sIL-6R increased from baseline (Table 3).

**Table 3:** Pharmacodynamic parameters (PD analysis set)

	ANC (× 10^6^/mL)		CRP (mg × dL)		sIL-6R (pg/mL)	
	RGB-19	Tocilizumab	RGB-19	Tocilizumab	RGB-19	Tocilizumab
Baseline						
n	180	185	180	185	180	185
Mean (SD)	5.59 (2.32)	5.48 (2.07)	2.05 (2.27)	2.01 (2.55)	31,910.0 (9089.9)	31,851.4 (8477.8)
Median	5.30	5.10	1.29	1.15	31,200	30,300
25th percentile-75th percentile	3.75-6.85	4.00-6.40	0.51-2.86	0.45-2.50	25,050-36,150	24,800-38,400
Week 12						
n	175	181	175	181	175	181
Mean (SD)	3.16 (1.73)	3.13 (2.16)	0.07 (0.33)	0.15 (0.89)	356,645.7 (131,256.8)	344,587.8 (134,949.5)
Median	2.80	2.60	0.01	0.01	334,000	327,000
25th percentile-75th percentile	1.90-3.80	1.90-3.70	0.01-0.02	0.01-0.02	259,000-449,000	245,000-454,000
Week 24						
n	170	172	170	172	170	172
Mean (SD)	3.09 (1.73)	3.08 (1.65)	0.07 (0.38)	0.10 (0.79)	425,661.2 (127,006.4)	413,584.9 (129,054.3)
Median	2.65	2.70	0.01	0.01	421,000	409,500
25th percentile-75th percentile	2.00-3.60	2.00-3.80	0.01-0.02	0.01-0.02	336,000-507,000	336,000-491,000
Week 52						
n	165	164	165	165	165	165
Mean (SD)	3.04 (1.82)	2.80 (1.62)	0.04 (0.22)	0.03 (0.11)	449,207.9 (109,312.3)	436,312.7 (106,371.5)
Median	2.60	2.45	0.01	0.01	445,000	436,000
25th percentile-75th percentile	2.00-3.50	1.80-3.30	0.01-0.02	0.01-0.02	384,000-519,000	373,000-502,000

ANC, absolute neutrophil count; CRP, C-reactive protein; PD, pharmacodynamic; sIL-6R, soluble IL-6 receptor.

### Safety

A total of 337 (91.6%) participants experienced 1468 TEAEs (Table 4); 164 (90.1%) participants experienced 695 TEAEs with RGB-19 treatment and 173 (93.0%) participants experienced 773 TEAEs with tocilizumab treatment. The most commonly reported TEAE was nasopharyngitis (RGB-19: 32 [17.6%] participants; tocilizumab: 42 [22.6%] participants) (Supplementary Table S3).

**Table 4:** Summary of adverse events (SAS)

	Treatment					
	RGB-19 (N = 182)		Tocilizumab (N = 186)		Total (N = 368)	
	n (%)	e	n (%)	e	n (%)	e
TEAEs	164 (90.1)	695	173 (93.0)	773	337 (91.6)	1468
ADRs ADRs in ≥5% of participants overall	100 (54.9)	235	109 (58.6)	253	209 (56.8)	488
White blood cell count decreased	17 (9.3)	22	24 (12.9)	35	41 (11.1)	57
Nasopharyngitis	12 (6.6)	18	12 (6.5)	13	24 (6.5)	31
Liver function test increased	9 (4.9)	9	10 (5.4)	10	19 (5.2)	19
Hepatic function abnormal	8 (4.4)	8	11 (5.9)	12	19 (5.2)	20
Hepatic enzyme increased	8 (4.4)	10	11 (5.9)	12	19 (5.2)	22
Severe ADRs	2 (1.1)	2	9 (4.8)	11	11 (3.0)	13
ADRs resulting in death	0 (0.0)	0	0 (0.0)	0	0 (0.0)	0
Serious ADRs	3 (1.6)	3	9 (4.8)	12	12 (3.3)	15
ADRs resulting in discontinuation of IP administration	5 (2.7)	5	9 (4.8)	9	14 (3.8)	14
All AESIs	147 (80.8)	371	152 (81.7)	434	299 (81.3)	805
Notable AESIs by category						
Infections^a^	99 (54.4)	189	101 (54.3)	190	200 (54.3)	379
Hypersensitivity^b^	30 (16.5)	36	42 (22.6)	52	72 (19.6)	88
Hepatic function disorders^c^	56 (30.8)	70	66 (35.5)	82	122 (33.2)	152
Administration site reactions^d^	0 (0.0)	0	3 (1.6)	4	3 (0.8)	4

ADR, adverse drug reaction; AE, adverse event; AESI, adverse event of special interest; e, event; IP, investigational product; SAE, serious adverse event; SAS, safety analysis set; TEAE, treatment-emergent adverse event.

a Occurring in ≥2% of participants in any group: infections and infestations (nasopharyngitis, COVID-19, upper respiratory tract infection, pharyngitis, bronchitis, sinusitis, gastroenteritis, paronychia, influenza, and herpes zoster).

b Occurring in ≥2% of participants in any group: respiratory, thoracic, and mediastinal disorders (allergic rhinitis), skin and subcutaneous tissue disorders (eczema, rash, contact dermatitis, urticaria).

c Hepatobiliary disorders (hepatic function abnormal, hepatic steatosis, liver disorder, drug-induced liver injury).

d General disorders and administration site conditions (injection site pain, infusion site erythema, injection site swelling, infusion site swelling).

In the RGB-19 group, 6 (3.3%) participants experienced severe AEs compared with 17 (9.1%) participants in the tocilizumab group. There were 6 (3.3%) participants in the RGB-19 group who experienced an SAE vs 18 (9.7%) in the tocilizumab group.

A similar proportion of participants who received RGB-19 treatment experienced adverse drug reactions (ADRs) compared with tocilizumab (RGB-19: 100 [54.9%] participants; tocilizumab: 109 [58.6%] participants). Two (1.1%) participants in the RGB-19 group experienced severe ADRs compared with 9 (4.8%) in the tocilizumab group (Table 4). The most reported ADR was a decrease in white blood cell count (RGB-19: 17 [9.3%] participants vs tocilizumab: 24 [12.9%] participants). Three participants (1.6%) in the RGB-19 group and 12 participants (6.5%) in the tocilizumab group experienced an ADR of the preferred term ‘neutrophil count decreased’ (Supplementary Table S4).

There was 1 death in the tocilizumab group during the study due to a severe haemorrhagic shock event following a road traffic accident that started 161 days after the first administration of IP. The investigator considered this to be unrelated to the IP. There was a similar proportion of participants who experienced the AESI of infections (RGB-19: 99 [54.4%] participants; tocilizumab: 101 [54.3%] participants) and only 4 administration site reactions occurred in 3 participants (1.6%), all in the tocilizumab group.

### Immunogenicity

The incidence of treatment-emergent (treatment-induced or treatment-boosted) ADAs up to and including week 52 was low for both treatment groups (RGB-19: 2.2% [n = 4]; tocilizumab 4.3% [n = 8]). In total, 13 ADA-positive participants were also classified as NAb positive; however, 2 participants from each group were NAb positive at baseline (RGB-19 2.7% [n = 5]; tocilizumab 4.3% [n = 8]).

## DISCUSSION

This study demonstrated the equivalence in efficacy of RGB-19 and reference tocilizumab, with the difference between treatments in the mean CfB in DAS28-ESR falling within the prespecified equivalence margin after 12 weeks of therapy in participants with active RA. This similarity between the 2 treatments was supported by the secondary efficacy outcomes between groups, including similar ACR, EULAR, CDAI, and SDAI achievement/response rates to week 52. Improvements in DAS28-ESR and response rates over time have been shown previously in trials with tocilizumab in RA [[34], [35], [36], [37], [38]], though differences in study populations preclude direct comparison with previous studies. Additionally, serum drug concentration and PD outcomes (ANC, CRP, sIL-6 levels) were similar between groups to week 52. The results of this study further support previous results of the phase 1 study, which demonstrated PK equivalence and similar PD outcomes [32]. The equivalence between RGB-19 and reference tocilizumab demonstrated in this study supports RGB-19 as an effective biosimilar; RGB-19 therefore has the potential to improve treatment options and patient access to cheaper IL-6-blocking biologics for RA.

RGB-19 demonstrated a similar immunogenicity profile to tocilizumab, with a low proportion of ADA-positive participants in both treatment groups, posttreatment administration. ADA status had no impact on drug concentration, although these findings were based on a small overall number of participants who were ADA positive.

Safety profiles were considered to be similar between groups, with comparable incidences of TEAE/ADRs and ADAs. A similar proportion of participants in the RGB-19 group experienced ADRs compared with the tocilizumab group. The incidence of treatment-emergent ADAs was similarly low in the RGB-19 and tocilizumab treatment groups, with this between-group similarity being consistent with previous phase 1 study results [32]. The results of this study build upon a robust quality evaluation programme that demonstrated the functional similarity of RGB-19 to tocilizumab [39]. Additionally, this study is consistent with previous data that have shown tocilizumab has an acceptable safety profile in clinical [14,19,20,40] and real-world settings, impacting treatment decisions for patients with RA and other autoimmune conditions treated with tocilizumab [41].

The most commonly reported ADR for both treatment groups was a decrease in white blood cell count, which is consistent with the expected ADR profile for tocilizumab [6]. ANC decreased in both treatment groups, with levels decreasing after baseline and remaining low until the end of treatment. It has been well-established that anti-IL-6 therapy can result in changes to neutrophil counts [42], with similar reductions reported for both tocilizumab and its other biosimilars [6,43,44]. It should be noted that it has been shown in ex vivo studies of neutrophils from patients with RA that tocilizumab-associated neutropenia does not affect neutrophil function associated with host defence [45]. In line with this, the proportion of participants who experienced serious infections or infestations in this study was low (<3%), and this was consistent with previous reports in patients treated with tocilizumab [11,14].

IL-6 signalling is involved in multiple inflammatory diseases [1,46]. Indeed, tocilizumab is a humanised anti-human IL-6R antibody that has been licenced for use in a wide range of indications including (dependent on region): polymyalgia rheumatica, giant cell arteritis, adult-onset Still disease, Takayasu arteritis, cytokine-release syndrome, Castleman disease, and COVID-19 [2,8]. As a tocilizumab biosimilar, RGB-19 has the potential to be used in multiple diseases [47].

Tocilizumab can be administered in both IV and SC formulations. SC may be preferred to IV administration, due to the convenience of self-administration and the reduced delivery time [48,49]. Preferences for mode of delivery vary by region, but the dual availability of tocilizumab provides flexibility for patients and physicians [48,49]. This study compared IV delivery of the 2 treatments; the equivalence in PK between RGB-19 and tocilizumab delivered SC was demonstrated in the phase 1 study [32].

Limitations of this study included a patient population restricted to Japanese participants; however, these results are expected to be generalisable to other populations, as population PK analyses in patients with RA have demonstrated that age, gender, and ethnic origin had no effect on tocilizumab PK [6].

### Conclusions and implications

Overall, this study demonstrated equivalent efficacy and similar safety, serum drug concentration, PD, and immunogenicity outcomes for RGB-19 and tocilizumab in patients with active RA and an inadequate response to methotrexate. These results, supported by the PK equivalence, quality analysis equivalence, and functional equivalence findings, demonstrate that RGB-19 can provide equivalent outcomes to tocilizumab in clinical settings. As a proposed biosimilar, RGB-19 may provide a cheaper alternative to tocilizumab that can increase patient access to IL-6 blocking therapies.

## Competing interests

HM has received consulting fees from Mochida Pharmaceutical, and Nichi-Iko Pharmaceutical, and honoraria and/or lecture fees from Chugai Pharmaceutical, Daiichi Sankyo, and Eli Lilly. EC has received research grants, speaking fees, consultancies or honoraria from AbbVie, Bio-Cancer, Biocon, Biogen, Chugai Pharma, Eli Lilly, Fresenius Kai, Galapagos, Gedeon Richter, Gilead, Janssen, Pfizer, Sanofi, UCB and Viatris. PE has provided expert advice to AbbVie, Activa, AstraZeneca, BMS, Boehringer Ingelheim, Galapagos, Gilead, Immunovant, Janssen, Lilly and Novartis, and contributed to clinical trials of AbbVie, BMS, Lilly, Novartis, and Samsung. MO has received speaking fees and/or honoraria from Astellas, Eli Lilly and Company, GSK and Janssen. RD, KH-K, GD, and JK are employees of Gedeon Richter. YK and SM are employees of Mochida Pharmaceutical. GRB has received honoraria for lectures and consulting from Celltrion, Chugai, Fresenius, Gedeon Richter, and Sanofi. Given his role as editor-in-chief, GRB had no involvement in the peer review of this article and has no access to information regarding its peer review. Full responsibility for the editorial process for this article was delegated to another journal editor.

## Appendix Supplementary materials

Image, application 1

Image, application 2

Image, application 3

## Footnotes

Supplementary material associated with this article can be found in the online version at doi:10.1016/j.ero.2025.12.010.
