# Supplementary material for: Efficacy, safety, and immunogenicity of proposed biosimilar RGB-19 and tocilizumab intravenously administered to adults with active rheumatoid arthritis and an inadequate response to methotrexate: a phase 3, randomised study
Source: EULAR Rheumatol Open. 2026 Jan 27;2(1):155–65. doi: 10.1016/j.ero.2025.12.010 (PMC13292490; doi:10.1016/j.ero.2025.12.010)
Supplement: Supplementary file 1 [file mmc1.pdf]

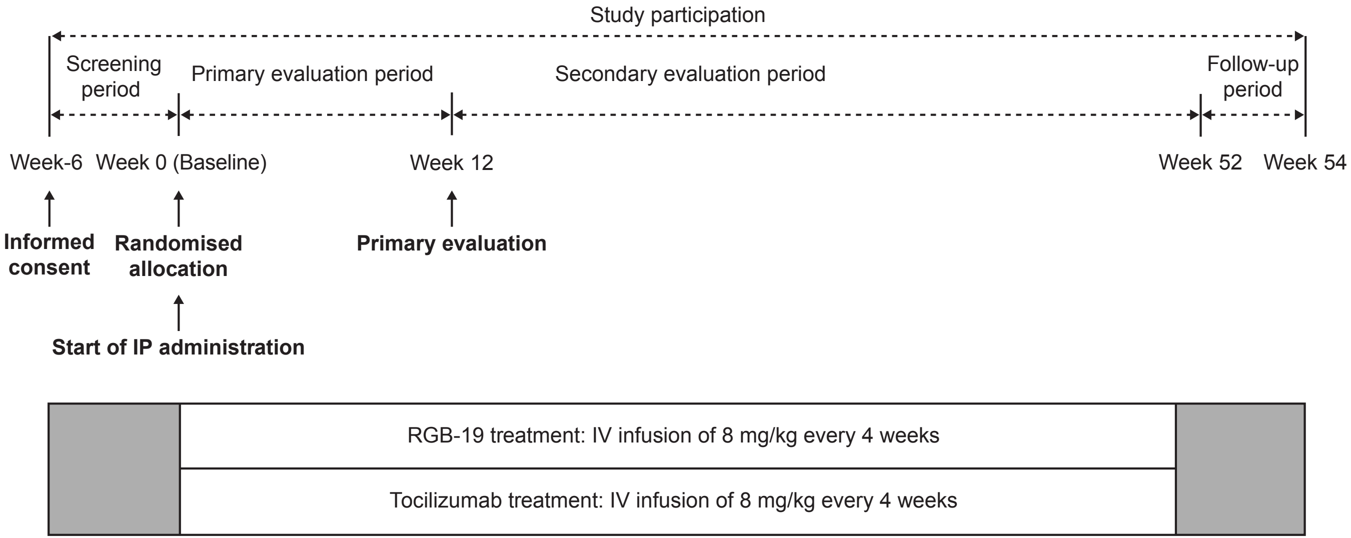
Study participation
Screening period
Primary evaluation period
Secondary evaluation period
Follow-up period
Week-6
Week 0 (Baseline)
Week 12
Week 52
Week 54
Informed consent
Randomised allocation
Primary evaluation
Start of IP administration
RGB-19 treatment: IV infusion of 8 mg/kg every 4 weeks
Tocilizumab treatment: IV infusion of 8 mg/kg every 4 weeks
