# Supplementary figures and images for: Efficacy, safety, and immunogenicity of proposed biosimilar RGB-19 and tocilizumab intravenously administered to adults with active rheumatoid arthritis and an inadequate response to methotrexate: a phase 3, randomised study
Source: EULAR Rheumatol Open. 2026 Jan 27;2(1):155–65. doi: 10.1016/j.ero.2025.12.010 (PMC13292490; doi:10.1016/j.ero.2025.12.010)

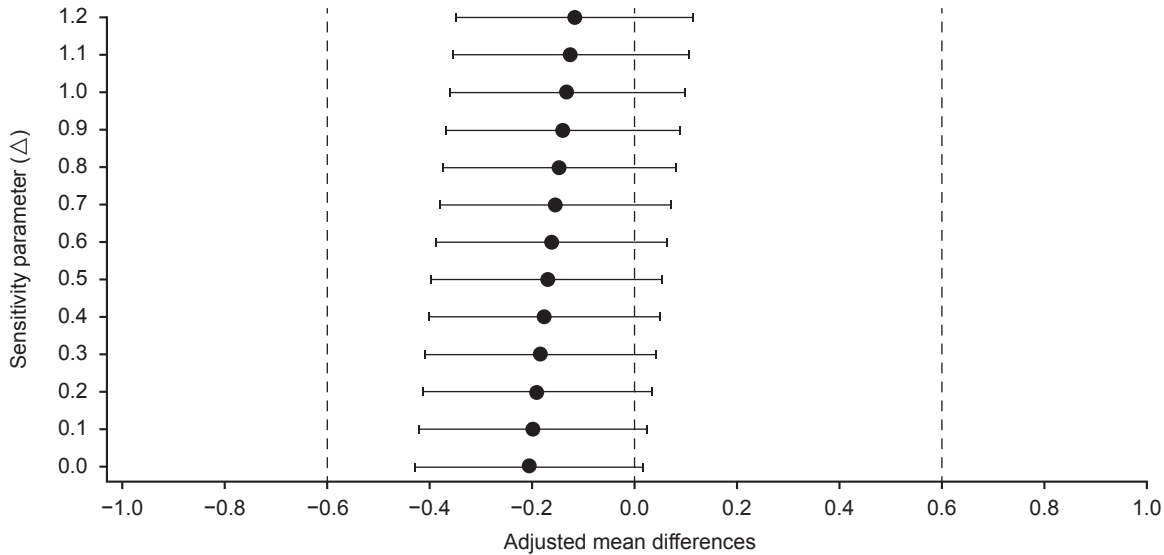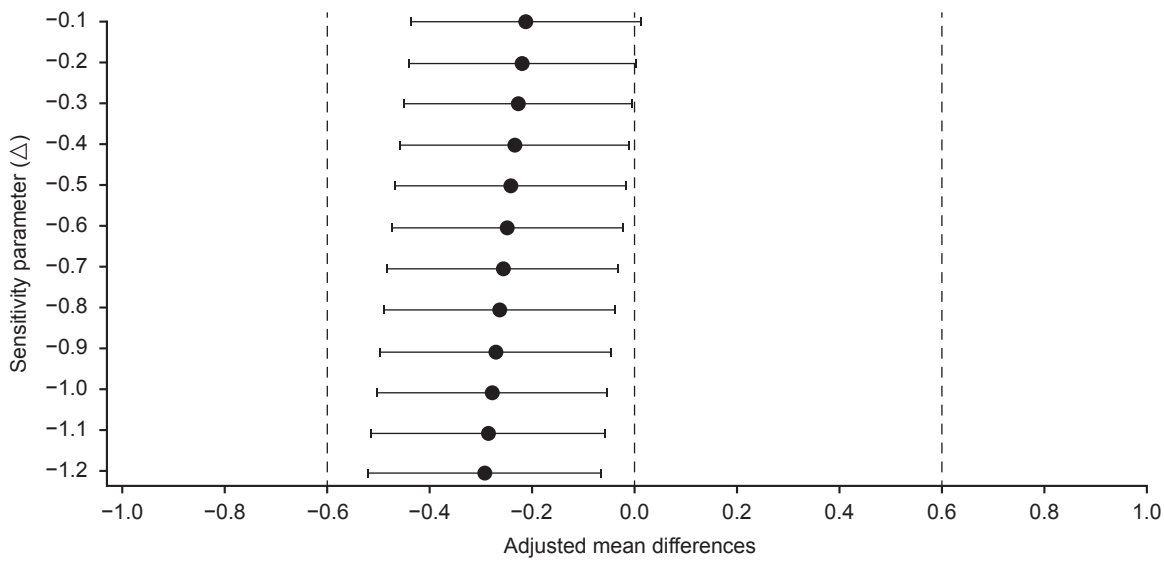

Supplement: Supplementary file 2 [file mmc2.pdf]
