# Supplementary material for: Efficacy, safety, and immunogenicity of proposed biosimilar RGB-19 and tocilizumab intravenously administered to adults with active rheumatoid arthritis and an inadequate response to methotrexate: a phase 3, randomised study
Source: EULAR Rheumatol Open. 2026 Jan 27;2(1):155–65. doi: 10.1016/j.ero.2025.12.010 (PMC13292490; doi:10.1016/j.ero.2025.12.010)
Supplement: Supplementary file 3 [file mmc3.docx]

# Efficacy, safety and immunogenicity of proposed biosimilar RGB-19 and tocilizumab intravenously administered to adults with active rheumatoid arthritis and an inadequate response to methotrexate: a Phase 3, randomised study

#

# Supplementary Material

## Supplementary Methods

### Full exclusion criteria

- Participants with a body weight of >100 kg or <30 kg at Screening or Baseline.
- Participants who, at the time of the study, had or had a history of any lymphoproliferative disease (e.g. chronic active Epstein–Barr virus infection), infection of prosthetic joint or significant secondary systemic disorders resulting from RA or autoimmune diseases other than RA. However, participants with Sjogren’s syndrome or chronic thyroiditis could be enrolled.
- Participants who, at the time of the study, had or had a history of any of the following diseases within 24 weeks before the joint assessment at Baseline: serious infectious disease (e.g. sepsis, bacterial pneumonia and nontuberculous mycobacteria); opportunistic infectious diseases (e.g. pneumocystis pneumonia and herpes zoster); chronic or recurrent infectious disease that was not suitable for this study in the opinion of the Investigators.
- Participants who, at Screening, had active tuberculosis or latent tuberculosis based on their clinical symptoms at the time of the study, a chest X-ray test and an interferon-γ release assay. However, participants with latent tuberculosis could be enrolled only if the participant could be treated in parallel with the study.
- Participants who, at Screening, had any seropositive result for hepatitis B surface (HBs) antigen, HBs antibody, hepatitis B core (HBc) antibody, hepatitis C virus antibody, or human immunodeficiency virus antigen and antibody tests. However, participants whose HBs antibody and/or HBc antibody test result was positive could be enrolled if their HBs antigen was negative and hepatitis B virus-DNA titre was <1.3 log IU/mL.
- Participants with diverticulitis.
- Participants who, at Screening, had any of the following laboratory test results: white blood cell count <3,500/mm3; lymphocyte count <500/mm3; neutrophil count <1,500/mm3; platelet count <100,000/mm3; aspartate aminotransferase >3 × upper limit of normal (ULN); alanine aminotransferase >3 × ULN; estimated glomerular filtration ratio <30 mL/min; blood beta-D-glucan positive.
- Participants who received treatment with 2 or more DMARDs or their biosimilar products. Drugs under development as biopharmaceuticals or biosimilar products for participants with RA (e.g. when administered as investigational products) are counted as biological DMARDs, but in the case of participation in clinical studies of these drugs, only placebo administered will not be counted as biological DMARDs. Biological DMARDs and their biosimilar products are counted together as one drug, regardless of the route of administration.
- Participants who received treatment with a biological DMARD or a biosimilar product within 12 weeks before the joint assessment at Baseline but within 16 weeks for participants who received ozoralizumab.
- Participants who ever received tocilizumab, any other IL-6 inhibitors or IL-6 receptor inhibitors, or Janus kinase inhibitors.
- Participants treated with csDMARDs (excluding oral MTX and leflunomide) within 4 weeks before the joint assessment at baseline.
- Participants treated with leflunomide within 12 weeks before the joint assessment at Baseline. However, participants who took colestyramine 8 g three times a day (8 g/day × tid) for 11 days and an additional wash-out period of more than 4 weeks from the discontinuation of leflunomide treatment, could be enrolled.
- Participants who received strong opioid analgesics within 4 weeks before the joint assessment at Baseline.
- Participants who had a surgical operation that could have affected the joints assessment (e.g. arthroplasty including synovectomy and artificial joint replacement) within 24 weeks or participants who had an arthrocentesis or intra-articular administration within 4 weeks before the joint assessment at Baseline.
- Participants whose dose/dosage of their regularly used oral or suppository non-steroidal anti-inflammatory drugs (NSAIDs)/analgesic drugs/corticosteroids was changed within 4 weeks before the joint assessment at Baseline. Here, regular treatment meant repeated administration of drugs in a constant dose and frequency. Adding new drugs as regular treatment was not allowed.
- Participants who used corticosteroids at >10 mg/day prednisolone equivalent dose within 4 weeks before the joint assessment at Baseline.
- Participants who used oral or suppository NSAIDs/analgesic drugs/corticosteroids temporarily within 24 hours before the joint assessment at Baseline. Temporary treatment meant any use other than repeated administration of drugs in a constant dose and frequency.
- Participants who used transdermal NSAIDs/analgesic drugs/corticosteroids (e.g. ointment, cream and patch) within 24 hours before the joint assessment at Baseline.
- Participants who used NSAIDs/analgesic drugs/corticosteroids by IV, intramuscular, intra-articular, or epidural administration within 4 weeks before the joint assessment at Baseline.
- Participants who received plasmapheresis and cytapheresis within 4 weeks before the joint assessment at Baseline.
- Participants treated with intra-articular administration of a drug containing hyaluronan as an active ingredient within 4 weeks before the joint assessment at Baseline.
- Participants treated with live viral or bacterial vaccines within 12 weeks before the joint assessment at Baseline.
- Participants treated with other IPs within 16 weeks before the date of signing Informed Consent.
- Participants who, at the time of the study, had any serious diseases such as cardiovascular, lung, liver, kidney, gastrointestinal, thyroid, haematological, psychoneurotic, or metabolic/electrolyte abnormality diseases (as judged by the Investigator, referring to Grade 3 or higher of the Common Terminology Criteria for Adverse Events v5.0).
- Participants who, at the time of the study, had or had the history of malignant tumours (including haematological malignancies) within 5 years before Baseline. However, participants who recovered from stage 0 non-invasive basal or squamous cell carcinoma of the skin or cervical cancer could be enrolled even if they were previously diagnosed within 5 years before Baseline.
- Participants who were pregnant, possibly pregnant, and/or breastfeeding; female participants who planned to become pregnant during the study period, or male participants who wished their partners became pregnant during the study period.
- Participants with prior history of anaphylaxis or hypersensitivity to monoclonal antibody-based therapies or any components of the IP’s formulations.
- Participants who, at the time of the study, had or had history of alcohol and/or drug abuse.
- Participants who were not suitable for this study for any other reason in the opinion of the investigator(s).

### Sensitivity parameters

- For the tipping point analysis, the same analysis model used for the primary analysis was used to calculate the difference between groups and the two-sided 95% CI for each value of sensitivity parameter (Δ).
- Using the same multiple imputation procedure as that of the primary analysis, to handle missing data in DAS28-ESR, only Δ was added to all imputed values in the RGB-19 treatment group.
- The primary focus of the study was the sensitivity parameters in the direction of worsening (0≤∆≤1.2). The sensitivity parameters in the direction of improvement (-1.2≤∆<0) were also considered.

### List of Institutional Review Boards

| Study centre number | Address |
| --- | --- |
| 1 | NAKAMEGURO ATLAS CLINIC IRB  1-26-1 Kamimeguro, Meguro-ku, Tokyo,  153-0051, Japan |
| 2 | Katayama Orthopedic Rheumatology  Clinic Institutional Review Board  4-5-17 Toyooka 13-jo, Asahikawa,  Hokkaido, 078-8243, Japan |
| 3 | Nihonbashi Sakura Clinic IRB  5F Inamura Building, 1-9-2  Nihonbashikayaba-cho, Chuo-ku, Tokyo,  103-0025, Japan |
| 4 | NAKAMEGURO ATLAS CLINIC IRB  1-26-1 Kamimeguro, Meguro-ku, Tokyo,  153-0051, Japan |
| 5 | NAKAMEGURO ATLAS CLINIC IRB  1-26-1 Kamimeguro, Meguro-ku, Tokyo,  153-0051, Japan |
| 6 | National Hospital Organization  Sagamihara National Hospital Institutional Review Board  18-1 Sakuradai, Minami-ku, Sagamihara,  Kanagawa, 252-0392, Japan |
| 7 | Fukui General Hospital IRB  58-16-1 Egami-cho, Fukui-shi, Fukui, 910-  8561, Japan |
| 8 | Social Medical Care Corporation Hoseikai  Marunouchi Hospital Institutional  Review Board  1-7-45 Nagisa, Matsumoto-shi, Nagano,  390-8601, Japan |
| 9 | NAKAMEGURO ATLAS CLINIC IRB  1-26-1 Kamimeguro, Meguro-ku, Tokyo,  153-0051, Japan |
| 10 | NAKAMEGURO ATLAS CLINIC IRB  1-26-1 Kamimeguro, Meguro-ku, Tokyo,  153-0051, Japan |
| 11 | Hiroshima University hospital IRB  1-2-3 Kasumi, Minami-ku, Hiroshima-shi,  Hiroshima, 734-0037, Japan |
| 12 | NAKAMEGURO ATLAS CLINIC IRB  1-26-1 Kamimeguro, Meguro-ku, Tokyo,  153-0051, Japan |
| 13 | Kitakyushu City Hospital Organization  Institutional Review Board  1-35 Furusenba-cho, Kitakyushu,  Kokurakita-ku, Fukuoka, 802-0082, Japan |
| 14 | Sasebo Chuo Hospital IRB  15 Yamato-cho, Sasebo-shi, Nagasaki,  857-1195, Japan |
| 15 | Nihonbashi Sakura Clinic IRB  5F Inamura Building, 1-9-2  Nihonbashikayaba-cho, Chuo-ku, Tokyo,  103-0025, Japan |
| 16 | Kumamoto Shinto General Hospital IRB  3-2-65 Oe, Chuo-ku, Kumamoto-shi,  Kumamoto, 862-8655, Japan |
| 17 | NAKAMEGURO ATLAS CLINIC IRB  1-26-1 Kamimeguro, Meguro-ku, Tokyo,  153-0051, Japan |
| 18 | Hokkaido Medical Center for Rheumatic  Diseases  3-1-45 Kotoni 1-jo, Nishi-ku, Sapporo-shi,  Hokkaido, 063-0811, Japan |
| 19 | Sugiura Clinic Institutional Review Board  4-4-16-301 Hon-cho, Kawaguchi-shi,  Saitama, 332-0012, Japan |
| 20 | Sugiura Clinic Institutional Review Board  4-4-16-301 Hon-cho, Kawaguchi-shi,  Saitama, 332-0012, Japan |
| 21 | NAKAMEGURO ATLAS CLINIC IRB  1-26-1 Kamimeguro, Meguro-ku, Tokyo,  153-0051, Japan |
| 22 | Sugiura Clinic Institutional Review Board  4-4-16-301 Hon-cho, Kawaguchi-shi,  Saitama, 332-0012, Japan |
| 23 | Review Board of Human Rights and Ethics  for Clinical Studies Institutional Review  Board  2-2-1, Kyobashi, Chuo-ku, Tokyo, 104-  0031, Japan |
| 24 | Inoue Hospital IRB  55 Tori-machi, Takasaki-shi, Gunma, 370-  0053, Japan |
| 25 | Sugiura Clinic Institutional Review Board  4-4-16-301 Hon-cho, Kawaguchi-shi,  Saitama, 332-0012, Japan |
| 26 | Sugiura Clinic Institutional Review Board  4-4-16-301 Hon-cho, Kawaguchi-shi,  Saitama, 332-0012, Japan |
| 27 | Doujin Memorial Medical Foundation,  Meiwa Hospital IRB  1-18 Kandasuda-cho,Chiyoda-ku, 101-  0041, Tokyo |
| 28 | Chibaken Saiseikai Narashino Hospital  IRB  1-1-1 Izumi-cho, Narashino-shi, Chiba,  1275-8580, Japan |
| 29 | Review Board of Human Rights and Ethics  for Clinical Studies Institutional Review Board  2-2-1 Kyobashi, Chuo-ku, Tokyo, 104-  0031, Japan |
| 30 | Nihonbashi Sakura Clinic IRB  5F Inamura Building, 1-9-2  Nihonbashikayaba-cho, Chuo-ku, Tokyo,  103-0025, Japan |
| 31 | NAKAMEGURO ATLAS CLINIC IRB  1-26-1 Kamimeguro, Meguro-ku, Tokyo,  153-0051, Japan |
| 32 | Yokohama City University Public  University Corporation Yokohama City  University Citizens General Medical  Center Clinical Trial Review Committee  4-57 Urafune-cho, Minami-ku,  Yokohama-shi, Kanagawa, 232-0024,  Japan |
| 33 | Sugiura Clinic Institutional Review Board  4-4-16-301 Hon-cho, Kawaguchi-shi,  Saitama, 332-0012, Japan |
| 34 | Nagaoka Red Cross Hospital Institutional  Review Board  2-297-1 Senshu, Nagaoka-shi, Niigata,  940-2085, Japan |
| 35 | Niigata Rheumatic Center IRB  1-2-8 Hon-cho, Shibata-shi, Niigata, 957-  0054, Japan |
| 36 | Fukui General Hospital IRB  58-16-1 Egami-cho, Fukui-shi, Fukui, 910-  8561, Japan |
| 37 | Sugiura Clinic Institutional Review Board  4-4-16-301 Hon-cho, Kawaguchi-shi,  Saitama, 332-0012, Japan |
| 38 | Sugiura Clinic Institutional Review Board  4-4-16-301 Hon-cho, Kawaguchi-shi,  Saitama, 332-0012, Japan |
| 39 | Review Board of Human Rights and Ethics  for Clinical Studies Institutional Review  Board  2-2-1 Kyobashi, Chuo-ku, Tokyo, 104-  0031, Japan |
| 40 | Hamamatsu Clinical Research Network  Institutional Review Board  2-12-12 Sumiyoshi, Naka-ku, Hamamatsushi,  Shizuoka, 430-8558, Japan |
| 41 | Toyohashi Municipal Hospital Institutional  Review Board  50 Hachikenishi, Aotake-cho, Toyohashishi,  Aichi, 441-8570, Japan |
| 42 | Review Board of Human Rights and Ethics  for Clinical Studies Institutional Review  Board  2-2-1 Kyobashi, Chuo-ku, Tokyo, 104-  0031, Japan |
| 43 | National Hospital Organization Nagoya  Medical Center Institutional Review Board  4-1-1 Sannomaru, Naka-ku, Nagoya-shi,  Aichi, 460-0001, Japan |
| 44 | Review Board of Human Rights and Ethics  for Clinical Studies Institutional Review  Board  2-2-1 Kyobashi, Chuo-ku, Tokyo, 104-  0031, Japan |
| 45 | NAKAMEGURO ATLAS CLINIC IRB  1-26-1 Kamimeguro, Meguro-ku, Tokyo,  153-0051, Japan |
| 46 | Nihonbashi Sakura Clinic IRB  5F Inamura Building, 1-9-2  Nihonbashikayaba-cho, Chuo-ku, Tokyo,  103-0025, Japan |
| 47 | Matsubara Mayflower Hospital  Institutional Review Board  944-25 Fujita, Kato-shi, Hyogo, 673-1462,  Japan |
| 48 | Doujin Memorial Medical Foundation,  Meiwa Hospital IRB  1-18 Kandasuda-cho, Chiyoda-ku, Tokyo,  101-0041, Japan |
| 49 | Matsubara Mayflower Hospital  Institutional Review Board  944-25, Fujita, Kato-shi, Hyogo, 673-  1462, Japan |
| 50 | NAKAMEGURO ATLAS CLINIC IRB  1-26-1 Kamimeguro, Meguro-ku, Tokyo,  153-0051, Japan |
| 51 | Review Board of Human Rights and Ethics  for Clinical Studies Institutional Review  Board  2-2-1 Kyobashi, Chuo-ku, Tokyo, 104-  0031, Japan |
| 52 | Sugiura Clinic Institutional Review Board  4-4-16-301 Hon-cho, Kawaguchi-shi,  Saitama, 332-0012, Japan |
| 53 | Sanuki Municipal Hospital Institutional  Review Board  387-1 Ishidahigashikou, Sanga-cho,  Sanuki-shi, Kagawa, 769-2393, Japan |
| 54 | Sugiura Clinic Institutional Review Board  4-4-16-301 Hon-cho, Kawaguchi-shi,  Saitama, 332-0012, Japan |
| 55 | Review Board of Human Rights and Ethics  for Clinical Studies Institutional Review  Board  2-2-1 Kyobashi, Chuo-ku, Tokyo, 104-  0031, Japan |
| 56 | Institutional Review Board of  Hamanomachi Hospital  3-3-1 Nagahama, Chuo-ku, Fukuoka-shi,  Fukuoka, 810-8539, Japan |
| 57 | St. Mary's Hospital Institutional Review  Board  422 Tsubukuhon-machi, Kurume-shi,  Fukuoka, 830-8543, Japan |
| 58 | Aso Iizuka Hospital Institutional Review  Board  3-83 Yoshio-cho, Iizuka-shi, Fukuoka,  820-8505, Japan |
| 59 | Review Board of Human Rights and Ethics  for Clinical Studies Institutional Review  Board  2-2-1 Kyobashi, Chuo-ku, Tokyo, 104-  0031, Japan |
| 60 | Kyushu University Hospital Institutional  Review Board  3-1-1, Maidashi, Higasi-ku, Fukuoka-shi,  Fukuoka, 812-8582, Japan |
| 61 | Kyushu Rosai Hospital Institutional  Review Board  1-1, Sonekitamachi, Kokuraminami-ku,  Kitakyushu-shi, Fukuoka, 800-0296, Japan |
| 62 | Nihonbashi Sakura Clinic IRB  5F Inamura Building, 1-9-2  Nihonbashikayaba-cho, Chuo-ku, Tokyo,  103-0025, Japan |
| 63 | Nihonbashi Sakura Clinic IRB  5F Inamura Building, 1-9-2  Nihonbashikayaba-cho, Chuo-ku, Tokyo,  103-0025, Japan |
| 64 | Nihonbashi Sakura Clinic IRB  5F Inamura Building, 1-9-2  Nihonbashikayaba-cho, Chuo-ku, Tokyo,  103-0025, Japan |
| 65 | Nagasaki University Hospital Institutional  Review Board  1-7-1 Sakamoto, Nagasaki-shi, Nagasaki,  852-8501, Japan |
| 66 | NAKAMEGURO ATLAS CLINIC IRB  1-26-1 Kamimeguro, Meguro-ku, Tokyo,  153-0051, Japan |
| 67 | Katayama Orthopedic Rheumatology  Clinic Institutional Review Board  4-5-17 Toyooka 13-jo, Asahikawa-shi,  Hokkaido, 078-8243, Japan |
| 68 | Nihonbashi Sakura Clinic IRB  5F Inamura Building, 1-9-2  Nihonbashikayaba-cho, Chuo-ku, Tokyo,  103-0025, Japan |
| 69 | Sugiura Clinic Institutional Review Board  4-4-16-301 Hon-cho, Kawaguchi-shi,  Saitama, 332-0012, Japan |
| 70 | Sugiura Clinic Institutional Review Board  4-4-16-301 Hon-cho, Kawaguchi-shi,  Saitama, 332-0012, Japan |
| 71 | Nihonbashi Sakura Clinic IRB  5F Inamura Building, 1-9-2  Nihonbashikayaba-cho, Chuo-ku, Tokyo,  103-0025, Japan |
| 72 | Sugiura Clinic Institutional Review Board  4-4-16-301 Hon-cho, Kawaguchi-shi,  Saitama, 332-0012, Japan |
| 73 | Nihonbashi Sakura Clinic IRB  5F Inamura Building, 1-9-2  Nihonbashikayaba-cho, Chuo-ku, Tokyo,  103-0025, Japan |
| 74 | Nihonbashi Sakura Clinic IRB  5F Inamura Building, 1-9-2  Nihonbashikayaba-cho, Chuo-ku, Tokyo,  103-0025, Japan |
| 75 | Nihonbashi Sakura Clinic IRB  5F Inamura Building, 1-9-2  Nihonbashikayaba-cho, Chuo-ku, Tokyo,  103-0025, Japan |
| 76 | Sugiura Clinic Institutional Review Board  4-4-16-301 Hon-cho, Kawaguchi-shi,  Saitama, 332-0012, Japan |
| 77 | Japanese Red Cross Nagasaki Genbaku  Hospital Institutional Review Board  3-15 Mori-machi, Nagasaki-shi, Nagasaki,  852-8511, Japan |
| 78 | Sugiura Clinic Institutional Review Board  4-4-16-301 Hon-cho, Kawaguchi-shi,  Saitama, 332-0012, Japan |
| 79 | Nihonbashi Sakura Clinic IRB  5F Inamura Building, 1-9-2  Nihonbashikayaba-cho, Chuo-ku, Tokyo,  103-0025, Japan |
| 80 | Institutional Review Board of Matsuyama  Red Cross Hospital  1 Bunkyo-cho, Matsuyama-shi, Ehime,  790-8524, Japan |
| 81 | National Hospital Organization  Chibahigashi National Hospital  Institutional Review Board  673 Nitona-cho, Chuo-ku, Chiba-shi,  Chiba, 260-8712, Japan |

### Supplementary Table 1 – Baseline concomitant diseases and administration history (FAS)

| **Characteristics** | **RGB-19**  **(N=182)** | **Tocilizumab (N=186)** | **Total**  **(N=368)** |
| --- | --- | --- | --- |
| Presence of concomitant disease, n (%) | 171 (94.0) | 169 (90.9) | 340 (92.4) |
| Infections and infestations | 16 (8.8) | 17 (9.1) | 33 (9.0) |
| Neoplasms benign, malignant and unspecified (incl cysts and polyps) | 7 (3.8) | 9 (4.8) | 16 (4.3) |
| Blood and lymphatic system disorders | 22 (12.1) | 22 (11.8) | 44 (12.0) |
| Immune system disorders | 33 (18.1) | 25 (13.4) | 58 (15.8) |
| Endocrine disorders | 18 (9.9) | 11 (5.9) | 29 (7.9) |
| Metabolism and nutrition disorders | 70 (38.5) | 62 (33.3) | 132 (35.9) |
| Psychiatric disorders | 11 (6.0) | 12 (6.5) | 23 (6.3) |
| Nervous system disorders | 16 (8.8) | 19 (10.2) | 35 (9.5) |
| Eye disorders | 25 (13.7) | 21 (11.3) | 46 (12.5) |
| Ear and labyrinth disorders | 2 (1.1) | 3 (1.6) | 5 (1.4) |
| Cardiac disorders | 9 (4.9) | 11 (5.9) | 20 (5.4) |
| Vascular disorders | 57 (31.3) | 59 (31.7) | 116 (31.5) |
| Respiratory, thoracic and mediastinal disorders | 41 (22.5) | 40 (21.5) | 81 (22.0) |
| Gastrointestinal disorders  Hepatobiliary disorders  Skin and subcutaneous tissue disorders  Musculoskeletal and connective tissue disorders  Renal and urinary disorders  Reproductive system and breast disorders  Congenital, familial and genetic disorders  General disorders and administration site conditions  Investigations  Injury, poisoning and procedural complications  Surgical and medical procedures | 39 (21.4)  35 (19.2)  16 (8.8)  66 (36.3)  7 (3.8)  18 (9.9)  1 (0.5)  3 (1.6)  14 (7.7)  1 (0.5)  0 (0.0) | 47 (25.3)  34 (18.3)  29 (15.6)  53 (28.5)  11 (5.9)  18 (9.7)  0 (0.0)  1 (0.5)  18 (9.7)  5 (2.7)  1 (0.5) | 86 (23.4)  69 (18.8)  45 (12.2)  119 (32.3)  18 (4.9)  36 (9.8)  1 (0.3)  4 (1.1)  32 (8.7)  6 (1.6)  1 (0.3) |
| Administration history, n (%) |  |  |  |
| Any biological DMARDs | 38 (20.9) | 31 (16.7) | 69 (18.8) |
| TNFα-inhibitors | 28 (15.4) | 27 (14.5) | 55 (14.9) |
| Etanercept | 13 (7.1) | 9 (4.8) | 22 (6.0) |
| Ozoralizumab | 2 (1.1) | 6 (3.2) | 8 (2.2) |
| Infliximab | 3 (1.6) | 3 (1.6) | 6 (1.6) |
| Adalimumab | 4 (2.2) | 2 (1.1) | 6 (1.6) |
| Golimumab | 3 (1.6) | 2 (1.1) | 5 (1.4) |
| Certolizumab Pegol | 3 (1.6) | 1 (0.5) | 4 (1.1) |
| Infliximab Biosimilar 1 | 0 (0.0) | 2 (1.1) | 2 (0.5) |
| Etanercept Biosimilar 1 | 0 (0.0) | 2 (1.1) | 2 (0.5) |
| Monoclonal Antibodies |  |  |  |
| Otilimab | 7 (3.8) | 4 (2.2) | 11 (3.0) |
| Selective Immunosuppressants |  |  |  |
| Abatacept | 3 (1.6) | 0 (0.0) | 3 (0.8) |

DMARDs, disease-modifying antirheumatic drugs; FAS, full analysis set; TNF, tumour necrosis factor.

### Supplementary Table 2 – Sensitivity analysis of change from baseline in DAS28-ESR at Week 12 (FAS)

| **Change from baseline in DAS28-ESR (Week 12)** | **RGB-19** | **Tocilizumab** |
| --- | --- | --- |
| Sensitivity parameter 1.2 △ |  |  |
| Adjusted mean^a^ | -3.54 | -3.43 |
| SE | 0.09 | 0.10 |
| Adjusted mean difference^b^ |  |  |
| Point estimate | -0.12 | |
| Two-sided 95% CI | -0.35, 0.11 | |
| Sensitivity parameter 1.1 △ |  | |
| Adjusted mean^a^ | -3.55 | -3.42 |
| SE | 0.09 | 0.10 |
| Adjusted mean difference^b^ |  | |
| Point estimate | -0.13 | |
| Two-sided 95% CI | -0.36, 0.10 | |
| Sensitivity parameter 1.0 △ |  |  |
| Adjusted mean^a^ | -3.56 | -3.42 |
| SE | 0.09 | 0.10 |
| Adjusted mean difference^b^ |  |  |
| Point estimate | -0.13  -0.36, 0.10 | |
| Two-sided 95% CI |  |  |
| Sensitivity parameter 0.9 △ |  |  |
| Adjusted mean^a^ | -3.56 | -3.42 |
| SE | 0.09 | 0.09 |
| Adjusted mean difference^b^ |  |  |
| Point estimate | -0.14  -0.37, 0.09 | |
| Two-sided 95% CI |  |  |
| Sensitivity parameter 0.8 △ |  |  |
| Adjusted mean^a^ | -3.57 | -3.42 |
| SE | 0.09 | 0.09 |
| Adjusted mean difference^b^ |  |  |
| Point estimate | -0.15  -0.38, 0.08 | |
| Two-sided 95% CI |  |  |
| Sensitivity parameter 0.7 △ |  |  |
| Adjusted mean^a^ | -3.58 | -3.42 |
| SE | 0.09 | 0.09 |
| Adjusted mean difference^b^ |  |  |
| Point estimate | -0.16  -0.38, 0.07 | |
| Two-sided 95% CI |  |  |
| Sensitivity parameter 0.6 △ |  |  |
| Adjusted mean^a^ | -3.58 | -3.42 |
| SE | 0.09 | 0.09 |
| Adjusted mean difference^b^ |  |  |
| Point estimate | -0.16  -0.39, 0.06 | |
| Two-sided 95% CI |  |  |
| Sensitivity parameter 0.5 △ |  |  |
| Adjusted mean^a^ | -3.59 | -3.42 |
| SE | 0.09 | 0.09 |
| Adjusted mean difference^b^ |  |  |
| Point estimate | -0.17  -0.40, 0.05 | |
| Two-sided 95% CI |  |  |
| Sensitivity parameter 0.4 △ |  |  |
| Adjusted mean^a^ | -3.59 | -3.42 |
| SE | 0.09 | 0.09 |
| Adjusted mean difference^b^ |  |  |
| Point estimate | -0.18  -0.40, 0.05 | |
| Two-sided 95% CI |  |  |
| Sensitivity parameter 0.3 △ |  |  |
| Adjusted mean^a^ | -3.60 | -3.42 |
| SE | 0.09 | 0.09 |
| Adjusted mean difference^b^ |  |  |
| Point estimate | -0.18  -0.41, 0.04 | |
| Two-sided 95% CI |  |  |
| Sensitivity parameter 0.2 △ |  |  |
| Adjusted mean^a^ | -3.61 | -3.41 |
| SE | 0.09 | 0.09 |
| Adjusted mean difference^b^ |  |  |
| Point estimate | -0.19  -0.42, 0.03 | |
| Two-sided 95% CI |  |  |
| Sensitivity parameter 0.1 △ |  |  |
| Adjusted mean^a^ | -3.61 | -3.41 |
| SE | 0.09 | 0.09 |
| Adjusted mean difference^b^ |  |  |
| Point estimate | -0.20  -0.42, 0.02 | |
| Two-sided 95% CI |  |  |
| Sensitivity parameter 0.0 △ |  |  |
| Adjusted mean^a^ | -3.62 | -3.41 |
| SE | 0.09 | 0.09 |
| Adjusted mean difference^b^ |  |  |
| Point estimate | -0.21  -0.43, 0.02 | |
| Two-sided 95% CI |  |  |
| Sensitivity parameter -0.1 △ |  |  |
| Adjusted mean^a^ | -3.63 | -3.41 |
| SE | 0.09 | 0.09 |
| Adjusted mean difference^b^ |  |  |
| Point estimate | -0.21  -0.44, 0.01 | |
| Two-sided 95% CI |  |  |
| Sensitivity parameter -0.2 △ |  |  |
| Adjusted mean^a^ | -3.63 | -3.41 |
| SE | 0.09 | 0.09 |
| Adjusted mean difference^b^ |  |  |
| Point estimate | -0.22  -0.44, 0.00 | |
| Two-sided 95% CI |  |  |
| Sensitivity parameter -0.3 △ |  |  |
| Adjusted mean^a^ | -3.64 | -3.41 |
| SE | 0.09 | 0.09 |
| Adjusted mean difference^b^ |  |  |
| Point estimate | -0.23 | |
| Two-sided 95% CI | -0.45, -0.01 | |
| Sensitivity parameter -0.4 △ |  |  |
| Adjusted mean^a^ | -3.64 | -3.41 |
| SE | 0.09 | 0.09 |
| Adjusted mean difference^b^ |  |  |
| Point estimate | -0.24 | |
| Two-sided 95% CI | -0.46, -0.01 | |
| Sensitivity parameter -0.5 △ |  |  |
| Adjusted mean^a^ | -3.65 | -3.41 |
| SE | 0.09 | 0.09 |
| Adjusted mean difference^b^ |  |  |
| Point estimate | -0.24  -0.47 -0.02 | |
| Two-sided 95% CI |  |  |
| Sensitivity parameter -0.6 △ |  |  |
| Adjusted mean^a^ | -3.66 | -3.41 |
| SE | 0.09 | 0.09 |
| Adjusted mean difference^b^ |  |  |
| Point estimate | -0.25  -0.48, -0.03 | |
| Two-sided 95% CI |  |  |
| Sensitivity parameter -0.7 △ |  |  |
| Adjusted mean^a^ | -3.66 | -3.40 |
| SE | 0.09 | 0.09 |
| Adjusted mean difference^b^ |  |  |
| Point estimate | -0.26 | |
| Two-sided 95% CI | -0.48, -0.03 | |
| Sensitivity parameter -0.8 △ |  |  |
| Adjusted mean^a^ | -3.67 | -3.40 |
| SE | 0.09 | 0.09 |
| Adjusted mean difference^b^ |  |  |
| Point estimate | -0.27 | |
| Two-sided 95% CI | -0.49, -0.04 | |
| Sensitivity parameter -0.9 △ |  |  |
| Adjusted mean^a^ | -3.68 | -3.40 |
| SE | 0.09 | 0.09 |
| Adjusted mean difference^b^ |  |  |
| Point estimate | -0.27 | |
| Two-sided 95% CI | -0.50, -0.05 | |
| Sensitivity parameter -1.0 △ |  |  |
| Adjusted mean^a^ | -3.68 | -3.40 |
| SE | 0.09 | 0.09 |
| Adjusted mean difference^b^ |  |  |
| Point estimate | -0.28 | |
| Two-sided 95% CI | -0.51, -0.05 | |
| Sensitivity parameter -1.1 △ |  |  |
| Adjusted mean^a^ | -3.69 | -3.40 |
| SE | 0.09 | 0.09 |
| Adjusted mean difference^b^ |  |  |
| Point estimate | -0.29 | |
| Two-sided 95% CI | -0.52, -0.06 | |
| Sensitivity parameter -1.2 △ |  |  |
| Adjusted mean^a^ | -3.69 | -3.40 |
| SE | 0.09 | 0.10 |
| Adjusted mean difference^b^ |  |  |
| Point estimate | -0.30  -0.52, -0.07 | |
| Two-sided 95% CI |  |  |

ANCOVA, analysis of covariance; CI, confidence interval; DAS28-ESR, disease activity score in 28 joints-erythrocyte sedimentation rate; DMARDs, disease-modifying antirheumatic drugs; FAS, full analysis set; SE, standard error.

^a^ANCOVA model included the treatment group and the administration history of biological DMARDs as factors, and DAS28-ESR score at baseline as the covariate; ^b^RGB-19 treatment group - tocilizumab treatment group.

### Supplementary Table 3 – Summary of AEs occurring in ≥2% of participants in either group (SAS)

|  | **Treatment** | | | |  | | |  |
| --- | --- | --- | --- | --- | --- | --- | --- | --- |
|  | **RGB-19 (N=182)** | | **Tocilizumab (N=186)** | | | **Total (N=368)** | | |
|  | **n (%)** | **e** | **n (%)** | **e** | | **n (%)** | **e** | |
| Nasopharyngitis | 32 (17.6) | 49 | 42 (22.6) | 63 | | 74 (20.1) | 112 | |
| Stomatitis | 35 (19.2) | 41 | 33 (17.7) | 54 | | 68 (18.5) | 95 | |
| White blood cell count decreased | 18 (9.9) | 23 | 26 (14.0) | 37 | | 44 (12.0) | 60 | |
| COVID-19 | 22 (12.1) | 22 | 15 (8.1) | 16 | | 37 (10.1) | 38 | |
| Liver function test increased | 16 (8.8) | 16 | 13 (7.0) | 13 | | 29 (7.9) | 29 | |
| Liver function test abnormal | 12 (6.6) | 17 | 13 (7.0) | 16 | | 25 (6.8) | 33 | |
| Hepatic enzyme increased | 10 (5.5) | 12 | 15 (8.1) | 16 | | 25 (6.8) | 28 | |
| Hepatic function abnormal | 9 (4.9) | 9 | 14 (7.5) | 15 | | 23 (6.3) | 24 | |
| Upper respiratory tract infection | 13 (7.1) | 19 | 8 (4.3) | 11 | | 21 (5.7) | 30 | |
| Dyslipidaemia | 9 (4.9) | 9 | 7 (3.8) | 7 | | 16 (4.3) | 16 | |
| Cough | 8 (4.4) | 9 | 8 (4.3) | 8 | | 16 (4.3) | 17 | |
| Pharyngitis | 8 (4.4) | 11 | 8 (4.3) | 8 | | 16 (4.3) | 19 | |
| Oropharyngeal pain | 8 (4.4) | 9 | 8 (4.3) | 11 | | 16 (4.3) | 20 | |
| Neutrophil count decreased | 3 (1.6) | 3 | 13 (7.0) | 22 | | 16 (4.3) | 25 | |
| Eczema | 6 (3.3) | 7 | 9 (4.8) | 10 | | 15 (4.1) | 17 | |
| Rash | 6 (3.3) | 6 | 9 (4.8) | 12 | | 15 (4.1) | 18 | |
| Bronchitis | 7 (3.8) | 9 | 7 (3.8) | 9 | | 14 (3.8) | 18 | |
| Dental caries | 6 (3.3) | 6 | 8 (4.3) | 8 | | 14 (3.8) | 14 | |
| Arthropod sting | 4 (2.2) | 5 | 9 (4.8) | 9 | | 13 (3.5) | 14 | |
| Sinusitis | 7 (3.8) | 7 | 5 (2.7) | 7 | | 12 (3.3) | 14 | |
| Hypertension | 6 (3.3) | 6 | 6 (3.2) | 6 | | 12 (3.3) | 12 | |
| Back pain | 6 (3.3) | 6 | 5 (2.7) | 6 | | 11 (3.0) | 12 | |
| Gastroenteritis | 4 (2.2) | 5 | 7 (3.8) | 7 | | 11 (3.0) | 12 | |
| Paronychia | 3 (1.6) | 4 | 8 (4.3) | 8 | | 11 (3.0) | 12 | |
| Constipation | 8 (4.4) | 10 | 2 (1.1) | 2 | | 10 (2.7) | 12 | |
| Blood triglycerides increased | 7 (3.8) | 12 | 3 (1.6) | 3 | | 10 (2.7) | 15 | |
| Platelet count decreased | 7 (3.8) | 10 | 3 (1.6) | 4 | | 10 (2.7) | 14 | |
| Headache | 4 (2.2) | 7 | 6 (3.2) | 6 | | 10 (2.7) | 13 | |
| Alanine aminotransferase increased | 3 (1.6) | 3 | 7 (3.8) | 8 | | 10 (2.7) | 11 | |
| Vomiting | 1 (0.5) | 1 | 9 (4.8) | 9 | | 10 (2.7) | 10 | |
| Influenza | 6 (3.3) | 6 | 3 (1.6) | 3 | | 9 (2.4) | 9 | |
| Dermatitis contact | 5 (2.7) | 5 | 4 (2.2) | 4 | | 9 (2.4) | 9 | |
| Nausea | 5 (2.7) | 6 | 4 (2.2) | 4 | | 9 (2.4) | 10 | |
| Diarrhoea | 3 (1.6) | 3 | 6 (3.2) | 6 | | 9 (2.4) | 9 | |
| Weight increased | 6 (3.3) | 7 | 2 (1.1) | 2 | | 8 (2.2) | 9 | |
| Herpes zoster | 5 (2.7) | 5 | 3 (1.6) | 3 | | 8 (2.2) | 8 | |
| Aspartate aminotransferase increased | 3 (1.6) | 3 | 5 (2.7) | 5 | | 8 (2.2) | 8 | |
| Contusion | 3 (1.6) | 3 | 5 (2.7) | 5 | | 8 (2.2) | 8 | |
| Hyperlipidaemia | 3 (1.6) | 3 | 5 (2.7) | 6 | | 8 (2.2) | 9 | |
| Urticaria | 2 (1.1) | 2 | 6 (3.2) | 6 | | 8 (2.2) | 8 | |
| Blood cholesterol increased | 1 (0.5) | 1 | 7 (3.8) | 8 | | 8 (2.2) | 9 | |
| Blood bilirubin increased | 4 (2.2) | 4 | 3 (1.6) | 3 | | 7 (1.9) | 7 | |
| Dizziness | 4 (2.2) | 4 | 3 (1.6) | 3 | | 7 (1.9) | 7 | |
| Ingrowing nail | 4 (2.2) | 4 | 3 (1.6) | 3 | | 7 (1.9) | 7 | |
| Wound | 4 (2.2) | 4 | 3 (1.6) | 3 | | 7 (1.9) | 7 | |
| Rheumatoid arthritis^a^ | 3 (1.6) | 3 | 4 (2.2) | 4 | | 7 (1.9) | 7 | |
| Abdominal discomfort | 2 (1.1) | 2 | 5 (2.7) | 6 | | 7 (1.9) | 8 | |
| Chillblains | 5 (2.7) | 5 | 1 (0.5) | 1 | | 6 (1.6) | 6 | |
| Abdominal pain | 2 (1.1) | 2 | 4 (2.2) | 5 | | 6 (1.6) | 7 | |
| Abdominal pain upper | 2 (1.1) | 2 | 4 (2.2) | 6 | | 6 (1.6) | 8 | |
| Rhinitis allergic | 2 (1.1) | 3 | 4 (2.2) | 4 | | 6 (1.6) | 7 | |
| Seasonal allergy | 2 (1.1) | 2 | 4 (2.2) | 4 | | 6 (1.6) | 6 | |
| Skin abrasion | 4 (2.2) | 4 | 1 (0.5) | 1 | | 5 (1.4) | 5 | |
| Asthma | 1 (0.5) | 2 | 4 (2.2) | 5 | | 5 (1.4) | 7 | |
| Periodontal disease | 0 (0.0) | 0 | 5 (2.7) | 5 | | 5 (1.4) | 5 | |
| Toothache | 4 (2.2) | 4 | 0 (0.0) | 0 | | 4 (1.1) | 4 | |
| Tenosynovitis | 0 (0.0) | 0 | 4 (2.2) | 4 | | 4 (1.1) | 4 | |

AE, adverse event; e, number of events; N, number of subjects; n, number of subjects reporting at least 1 TEAE within preferred term; SAS, safety analysis set in the primary evaluation and secondary evaluation periods; TEAE, treatment-emergent adverse event.
^a^Verbatim terms: worsening of rheumatoid arthritis, worsening of the primary disease, worsening of rheumatic gonarthritis on the left side, and acute on rheumatoid arthritis*.*

### Supplementary Table 4 – Summary of adverse drug reactions occurring in ≥2% of participants in either group (SAS)

|  | **Treatment** | | | |  | | |  |
| --- | --- | --- | --- | --- | --- | --- | --- | --- |
|  | **RGB-19 (N=182)** | | **Tocilizumab (N=186)** | | | **Total (N=368)** | | |
|  | **n (%)** | **e** | **n (%)** | **e** | | **n (%)** | **e** | |
| White blood cell count decreased | 17 (9.3) | 22 | 24 (12.9) | 35 | | 41 (11.1) | 57 | |
| Nasopharyngitis | 12 (6.6) | 18 | 12 (6.5) | 13 | | 24 (6.5) | 31 | |
| Liver function test increased | 9 (4.9) | 9 | 10 (5.4) | 10 | | 19 (5.2) | 19 | |
| Hepatic function abnormal | 8 (4.4) | 8 | 11 (5.9) | 12 | | 19 (5.2) | 20 | |
| Hepatic enzyme increased | 8 (4.4) | 10 | 11 (5.9) | 12 | | 19 (5.2) | 22 | |
| Neutrophil count decreased | 3 (1.6) | 3 | 12 (6.5) | 21 | | 15 (4.1) | 24 | |
| Stomatitis | 6 (3.3) | 7 | 8 (4.3) | 10 | | 14 (3.8) | 17 | |
| Liver function test abnormal | 6 (3.3) | 7 | 6 (3.2) | 7 | | 12 (3.3) | 14 | |
| Platelet count decreased | 7 (3.8) | 10 | 3 (1.6) | 4 | | 10 (2.7) | 14 | |
| COVID-19 | 6 (3.3) | 6 | 4 (2.2) | 4 | | 10 (2.7) | 10 | |
| Dyslipidaemia | 4 (2.2) | 4 | 4 (2.2) | 4 | | 8 (2.2) | 8 | |
| Alanine aminotransferase increased | 3 (1.6) | 3 | 5 (2.7) | 6 | | 8 (2.2) | 9 | |
| Aspartate aminotransferase increased | 3 (1.6) | 3 | 5 (2.7) | 5 | | 8 (2.2) | 8 | |
| Blood triglycerides increased | 5 (2.7) | 10 | 2 (1.1) | 2 | | 7 (1.9) | 12 | |
| Blood cholesterol increased | 1 (0.5) | 1 | 6 (3.2) | 7 | | 7 (1.9) | 8 | |
| Blood bilirubin increased | 4 (2.2) | 4 | 2 (1.1) | 2 | | 6 (1.6) | 6 | |
| Cough | 4 (2.2) | 5 | 2 (1.1) | 2 | | 6 (1.6) | 7 | |
| Herpes zoster | 4 (2.2) | 4 | 2 (1.1) | 2 | | 6 (1.6) | 6 | |
| Bronchitis | 2 (1.1) | 3 | 4 (2.2) | 5 | | 6 (1.6) | 8 | |
| Oropharyngeal pain | 4 (2.2) | 5 | 0 (0.0) | 0 | | 4 (1.1) | 5 | |

Includes all TEAEs for which a causal relationship with the investigational product cannot be ruled out.
ADR, adverse drug reaction; e, number of events; N, number of subjects; n, number of subjects reporting at least 1 ADR within preferred term; SAS, safety analysis set in the primary evaluation and secondary evaluation periods; TEAE, treatment-emergent adverse event.

### Supplementary Figure 1 – Study design

IV, intravenous.

### Supplementary Figure 2 – Forest plot for sensitivity analysis change from baseline in DAS28-ESR at Week 12 (FAS)

DAS28-ESR, disease activity score based on 28 joints with erythrocyte sedimentation rate; FAS, full analysis set.
